# Food environment and consumption of ultra-processed foods influencing food addiction in socially vulnerable women in Brazil

**DOI:** 10.1017/S1368980025100426

**Published:** 2025-06-03

**Authors:** Luiz Gonzaga Ribeiro Silva-Neto, Nassib Bezerra Bueno, Camila Aparecida Borges, Risia Cristina Egito de Menezes, André Eduardo da Silva Júnior, Thays Lane Ferreira dos Santos, Telma Maria de Menezes Toledo Florêncio

**Affiliations:** 1 Programa de Pós-Graduação em Nutrição, Escola Paulista de Medicina, Universidade Federal de São Paulo, R. Botucatu, 740 - Vila Clementino, São Paulo 04023-062, SP, Brasil; 2 Programa de Pós-Graduação em Nutrição, Faculdade de Nutrição, Universidade Federal de Alagoas, Avenida Lourival Melo Mota, s/n. Tabuleiro dos Martins, CEP: 57072- 900, Maceió, AL, Brasil; 3 Núcleo de Pesquisas Epidemiológicas em Nutrição e Saúde, Faculdade de Saúde Pública, Universidade de São Paulo, Av. Dr. Arnaldo, CEP - 01246-90, 715 - São Paulo, SP, Brasil

**Keywords:** Poverty, Consumer food environment, Food consumption, Food addiction

## Abstract

**Objective:**

This study aimed to evaluate the association between food addiction (FA), food environment and consumption of ultra-processed foods (UPF) in socially vulnerable women.

**Design:**

A population-based cross-sectional study was conducted in Favelas and Urban Communities of Maceió-Brazil. The availability of UPF in the food environment was evaluated through the audit of retailers with the support of the AUDITNOVA instrument. The women taking part in the study were interviewed, and a 24-hour food recall was used to assess the proportion of UPF in their diet. The modified Yale Food Addiction Scale 2.0 was also used to determine FA. Association analysis was performed using binary logistic regression and generalised estimation equations.

**Participants:**

1702 adult women of reproductive age (20–44 years) residents in Favelas and Urban Communities.

**Results:**

It was found that 14·6 % of the women had FA. The adjusted multivariate association analysis showed that the high availability of UPF in food retail increased the chance of women having FA by up to 47 % (*P*= 0·02 OR: 1·53; 95 % CI: (1·07, 2·18)). It was also possible to observe that the greater calorific contribution of UPF in the diet increased the chance of women presenting FA by up to 61 % (*P*< 0·01 OR: 1·39; 95 % CI: (1·48, 1·97)).

**Conclusions:**

The environment and what is available in it are associated with additive behaviour independent of individual factors, and UPF consumption increases the chance of FA. This demonstrates the need for changes in the food environment in Brazilian favelas, contributing to improving women’s health.

## Introduction

Food addiction (FA) is a behaviour that is similar to substance use disorder and is characterised by excessive consumption of processed, hyper-palatable, energy-dense foods that are high in sugar, saturated fat and/or Na, the main characteristic of ultra-processed foods (UPF)^(1)^. A systematic review carried out by Praxedes et al.^(2)^ identified a prevalence of around 20 % of FA in the population. This situation has attracted the attention of the scientific community.

This is due to the fact that UPF are widely available in the consumer’s food environment, especially when looking at the Brazilian reality, a situation that is even more aggravated in poorer regions^(3)^. It is known that the more exposed an individual is to a specific type of food, the greater the probability of the individual consuming it^(4)^. Because of the possible association between UPF consumption and the development of FA^(5)^, it is reasonable to assume that the food environment plays an important role by acting as a risk factor for the behaviour of FA.

This situation is highlighted when comparing low-income and high-income regions in Brazil, where it is possible to observe the greater availability of UPF in the food environment of consumers in low-income areas^(6)^. The greater availability and easy access to UPF have a direct impact on the food consumption of the world’s population, making this type of food increasingly part of their diet^(7)^. This situation arouses attention in Brazil since 19·7 % of the energies consumed by the Brazilian population come from UPF, and among women, this number increases to 20·3 %^(8)^.

Due to the higher palatability and lower price of this type of product^(9)^, the higher frequency of UPF consumption has been associated with an increased risk of body weight gain^(10)^, hypertension^(11)^, type 2 diabetes^(12)^ and cancer^(13)^, between individuals living in different countries. It may still be related to an increase in FA^(14)^.

Given that greater exposure to UPF can result in increased consumption of this food group, it is pertinent to infer that the food environment in which the population resides plays a significant role in UPF consumption and may also influence FA. However, further research into this possible relationship is essential, especially in socioeconomically vulnerable women.

There is a need to study this relationship in women better due to the high magnitude of FA in this population group^(2)^. This is also due to the relationship between maternal and child food consumption^(15)^, which explains the increasingly early consumption of UPF by children^(16)^.

Thus, this study aimed to analyse the association between FA, the food environment and the consumption of UPF in women living in a socially vulnerable situation in a capital city in the Northeast of Brazil.

## Methods

### Design and location of the study

This cross-sectional population-based study was conducted between October 2020 and May 2021. It sought to assess the food environment, food intake and FA, as well as other individual and socioeconomic characteristics of adult women of reproductive age living in the Favelas and Urban Communities of Maceió. Maceió is the capital of the state of Alagoas, located in the northeast region of Brazil, and has the lowest Human Development Index (0·721 - http://www.br.undp.org) among the region’s capitals.

The Brazilian Institute of Geography and Statistics (Institutio Brasileiro de Geografia e Estatística - IBGE) characterises Favelas and Urban Communities as places with a predominance of households with varying degrees of legal insecurity of tenure and at least one of the other criteria: absence or incomplete provision of public services; predominance of buildings, streetscapes and infrastructure that are usually self-produced or are guided by urban planning and construction parameters other than those defined by public bodies; location in areas with restrictions on occupation defined by environmental or urban planning legislation^(17)^.

### Size and selection of sample

Taking into account the estimate that there are 24 614 women aged between 20 and 44 years in the Favelas and Urban Communities of Maceió, having as the outcome of interest the overweight in Maceió, estimated at 52·6 % in females^(18)^, adopting a margin of error of 3 % and a CI of 99 %, it would be necessary to recruit at least 1710 women. The sample size calculation was done with the help of StatCalc v. 7.2.5.0 (Center for Disease Control, Atlanta, EUA).

**Figure 1.**
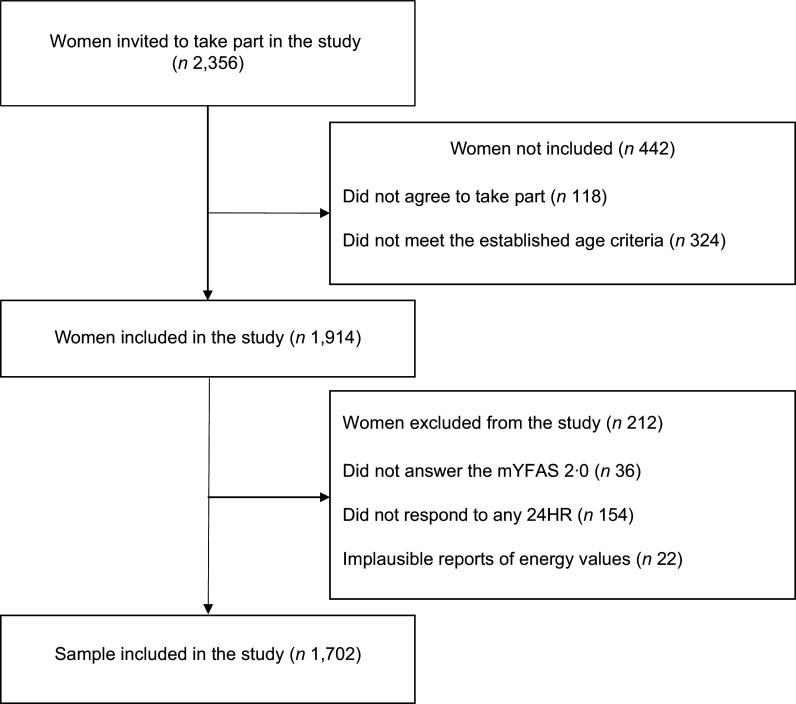
Flowchart including the women taking part in the study.

Data were collected from only one woman per household. A total of 2356 women were invited to take part in the study. After evaluating the inclusion and exclusion criteria described in Figure 1, 1702 women were included in the study. Women were recruited from forty Favelas and urban communities randomly selected (the flowchart for selecting Favelas and urban communities is available in the Supplementary material).

The sampling design was probabilistic and of the three-stage conglomerate type: 1) Favelas and Urban Communities, which were selected in a simple random proportional manner in each of the seven administrative regions of Maceió that were studied; 2) census sectors, one selected from each Favelas and Urban Communities, by simple random drawing and 3) streets, in each census sector evaluated a street was drawn for data collection from women.

All households on the selected street were visited, and when necessary, the surrounding ones until the sample corresponding to the location was complete. We included all households with at least one adult woman of reproductive age, between 20 and 44 years old. Pregnant women and women who had some disability that compromised their food intake or made it impossible to conduct the interview or understand the research questionnaires were not included.

### Data collection

Data were collected using a paper-based questionnaire at the homes of the women included in the study and the food retail evaluations. The collection team consisted of seven nutritionists and four undergraduate nutrition students. Two nutritionists supervised the collections and administered the questionnaires.

### Socio-demographic and health variables

Data were collected regarding age (years), race/skin colour (white, black, brown, Asian and Indigenous) and reported medical diagnosis of anxiety and depression. These variables were selected as potential confounding factors since they were already associated with FA in the Brazilian population^(19,20)^. Data were also collected regarding the monthly per capita family income to characterise the population, classifying it according to the cut-off points for poverty (poverty – US$ < 91·90; and out of poverty US$ ≥ 91·90. Values converted from Brazilian currency (Real) to US dollars, considering the average dollar exchange rate between October 2020 and May 2021 – R$ 5·43)^(21)^.

### Dietary assessment

A 24-hour dietary recall (24HR) was completed, corresponding to the day before the interview. In a sub-sample of at least 20 % of women, the 24HR was administered along with two other recalls by telephone up to 60 d after the first 24HR was administered to evaluate and correct intra-individual variability in consumption.

Of the three 24HR applied to the subsample, two corresponded to weekdays and one to the weekend. The 24HR was applied using the multiple pass method^(22)^. During the interviews, all food preparations were reported, and the participant was asked to describe each ingredient in the preparation.

The 24HR was analysed using the nutritional evaluation and prescription system Avanutri 4·0® (Três Rios, Rio de Janeiro, Brazil) considering three tables of the nutritional composition of foods, in the following order of priority: Brazilian Table of Food Composition^(23)^, Table of Food Composition^(24)^ and Tables of Nutritional Composition of Foods Consumed in Brazil^(25)^. Subsequently, the foods were classified according to their degree of processing to determine unprocessed or minimally processed foods, processed culinary ingredients, processed foods and UPF, considering the NOVA classification of foods proposed by Monteiro et al.^(26)^.

Two nutritionists coordinated the 24HR data collection. The other six nutritionists participated in the home visits for the 24HR application and were responsible for entering the food data into the software. Two different nutritionists double-entered each 24HR into the evaluation software. When any inconsistencies were identified, one of the supervising nutritionists corrected the 24HR into the evaluation software to validate the dietary data.

These data were estimated using the multiple source method^(27)^. This technique was applied to those women who had more than one 24HR. As previously mentioned, it was recommended that up to three 24HR be applied to at least 20 % of the sample. Of the women included in this study, 627 (36·8 %) had more than one 24HR.

Multiple source method is a statistical method used to estimate the usual food intake based on two or more consumption assessment measurements, such as 24HR data. The statistical algorithms of the multiple source method evaluate the intraindividual variation of intake. This method is characterised by a two-part shrinkage technique applied to the residuals of two regression models: one for the positive daily intake data and one for the probability of consumption^(27)^.

After checking the dietary data, we identified the participants with implausible energy values reports and excluded them from this evaluation. For this procedure, we adopted what Willett^(28)^ proposed: women who report a consumption below 500 kcal/d or above 3500 kcal/d were considered women with implausible reports. At the end of this procedure, the percentage of caloric contribution of the UPF was grouped into tertiles for statistical analysis.

### Food addiction

The Brazilian Portuguese translated and validated version of the modified Yale Food Addiction Scale 2.0 (mYFAS 2.0)^(20)^ was used to determine FA. The scale comprises thirteen questions, of which eleven represent symptoms of eating behaviour and two refer to clinical distress/impairment. Each question is answered according to the frequency with which it occurs, ranging from ‘never’ to ‘every day’, each of which has a limit for the criterion of the symptom to be met. In the end, the eleven symptoms are added together to create a symptom count score option. There is a threshold in each of them so that certain behaviour is considered a symptom^(29)^.

Positive cases of FA were considered those who reached the threshold for two or more symptoms and for at least one of the items that assess clinical distress. In addition, the number of positive symptoms can also be observed to ascertain not only the prevalence of FA in the population evaluated but also the average number of positive symptoms as a way of purchasing populations more generally.

### Consumer food environment

All formal and informal food retail^(30)^ identified within a 400-m buffer from the midpoint of the streets drawn for data collection from women in each Favelas and Urban Communities were audited. The total number of businesses audited was 624. The audit was conducted with the support of the AUDITNOVA instrument, validated for food retailers in Brazil, which assesses factors such as availability, price, variety and advertising strategies in food retail^(31)^. The entire audit process was conducted by the research team in person at the food retail.

From the data collected in the audit process, it was possible to calculate the availability of UPF for each of the businesses audited. For this procedure, all eighteen UPF available in AUDITNOVA were taken into account. The availability of UPF was also evaluated by a score ranging from 0 (no UPF) to 18 (presence of all audited UPF) and was standardised for a scale of 0–100 points, as used in a study by Serafim et al.^(6)^. The final mean score of each settlement was also stratified into tertiles, the first being considered low availability, the second medium and the third high availability.

### Spatial data

Geographic coordinates (latitude and longitude) were collected from all retailers audited through the Google Earth application v. 9.3.25.5 (Google, United States), positioned at a distance of 1 m from its main entrance. Subsequently, these data were entered into the software QGIS 3.16.15 (Open Source Geospatial Foundation, Chicago, United States)

A layer of buffers with a radius of 400 m was created, calculated from the midpoint of each street drawn for the beginning of the collections in each settlement. After superimposing this layer with another layer containing level data of the establishments, the average values of the UPF availability measure were calculated for each buffer, providing information on each Favelas and Urban Communities. Subsequently, these values were grouped into tertiles to perform the statistical analyses.

### Data analysis

Descriptive analyses were performed for the individuals’ characteristics and the food environment, with continuous variables presented as mean and sd and categorical variables as absolute and relative frequencies. Differences between proportions were compared according to the presence or absence of FA using a *t* test (for age) and a chi-square test (for categorical variables).

The outcome variable was the presence of FA. As exposure variables, we considered the availability of UPF in the food environment (values categorised into tertiles: low availability, medium availability and high availability) and the caloric contribution from UPF in the diet of women (values categorised into tertiles: low caloric contribution, medium caloric contribution and higher caloric contribution).

Association analysis was performed using binary logistic regression and generalised estimation equations. The OR and their respective 95 % CI estimated the association. The analyses were adjusted for the following variables used as confounding factors: age, race/skin colour, per capita income and previous medical diagnosis of anxiety and depression. The analyses were performed using the statistical software Jamovi Computer Software (Version 2.3, The jamovi project, Sydney, Australia).

## Results

The demographic and health characteristics are detailed in Table 1. The average age of women was 31·1 (7·9) years; 62·7 % self-declared as brown and 74·5 % lived in poverty situation. It was also possible to identify that 11·3 % and 4·7 % of women had a previous medical diagnosis of anxiety and depression, respectively. Regarding FA, 14·6 % of women had this condition, and the mean FA symptom count in the study population was 1·06 (1·9) symptoms.

**Table 1.** Socio-demographic and health characteristics of women in social vulnerability participating in the study. Maceió, 2020/2021 (*n* 1702)

	Total (*n* 1702)		With food addiction (*n* 248 – 14·6 %)		No food addiction (*n* 1454 – 85·4 %)		
Variables	*n*	%	*n*	%	*n*	%	*P*-value
Age							0·12
Average	31·1		31·2		30·4		
DP	7·9		8·0		7·1		
Race/skin color							
White	240	14·1	20	8·1	220	15·1	< 0·01
Black	257	15·1	46	18·5	211	14·5	
Brown	1067	62·7	155	62·5	912	62·7	
Asian	127	7·5	27	10·9	100	6·9	
Indigenous	11	0·6	0	0·0	11	0·8	
Monthly per capita family income^*^							0·84
US$ < 91·90	1268	74·5	186	75·0	1082	74·4	
US$ ≥ 91·90	434	25·5	62	25·0	372	25·6	
Reported medical diagnosis of anxiety							
No	1510	88·7	213	85·9	1297	89·6	0·13
Yes	192	11·3	35	14·1	157	10·8	
Reported medical diagnosis of depression							< 0·01
No	1622	95·3	228	91·9	1394	95·9	
Yes	80	4·7	20	8·1	60	4·1	

The modified Yale Food Addiction Scale 2.0 was used to evaluate food addiction.

* Variable classified as proposed by World Bank Mundial^(21)^ to determine the situation of poverty (US$ < 91·90); out of poverty (US$ ≥ 91·90). Values converted from reais to US dollars, considering the average dollar exchange rate between October 2020 and May 2021 – R$ 5·43.

Regarding food consumption, the average number of energies ingested at UPF was 886·3 (280·8) kcal. More information on food consumption is shown in Table 2. Regarding the food environment, the mean score of the UPF availability score was 41·7 (8·5) points; it was identified that the five UPF most available in the establishments were candy (87·5), sandwich cookies (87·3), 2-l soda (84·5), soft drink mix (80·0) and snacks (79·3) (Table 3).

**Table 2.** Dietary characteristics of women in social vulnerability participating in the study. Maceió, 2020/2021 (*n* 1702)

Variables	Mean	sd
Total energy intake	2624·8 kcal	499·9
Energies of fresh and minimally processed foods	1084·7 kcal	315·7
Energetic contribution of fresh and minimally processed foods	41·3 %	14·6
Energies of culinary ingredients	28·9 kcal	31·2
Energetic contribution of culinary ingredients	1·1 %	1·3
Energies of processed foods	624·9 kcal	189·4
Energetic contribution of processed foods	23·8 %	8·6
Energies of UPF	886·3 kcal	280·8
Energetic contribution from UPF	33·8 %	11·1

UPF, ultra-processed foods.

**Table 3.** Availability of ultra-processed foods in the consumer’s food environment. Maceió, 2020/2021 (*n* 624)

UPF availability	*n* ^*^	%
Score (points)		
Mean	41·7	
sd	8·4	
Hot dog	281	45·0
Pork sausage	191	30·6
Bread	97	15·6
Breakfast cereals	52	8·3
Snacks	495	79·3
Sandwich cookies	545	87·3
Ice cream	80	12·8
Chocolate	285	45·7
Candy	546	87·5
Soda can	371	59·5
2-L soda	527	84·5
Zero/light/diet soda	115	18·4
Néctar	73	11·7
Soft drink mix	499	80·0
Milk drink	180	28·9
Readymade pizza	42	6·7
Seasoning mix	422	67·6
Instant noodles	478	76·6

UPF, ultra-processed foods.

Through the audit of retailers with the support of the AUDITNOVA instrument^(31)^, the availability of UPF in the consumer’s food environment was assessed^(6)^.

* Number of establishments that had the UPF available.

The adjusted multivariate regression showed that women who lived in places with high availability of UPF in the food environment were up to 47 % more likely to have FA (*P*= 0·02 OR: 1·53; 95 % CI: (1·07, 2·18); Table 4). It was also possible to observe that the higher caloric contribution of UPF in the diet increased the chance of women having FA by up to 61 % (*P*< 0·01 OR: 1·39; 95 % CI: (1·48, 1·97); Table 4).

**Table 4.** Crude and adjusted OR (95 %CI) for food addiction according to the availability of UPF in the consumer’s food environment and caloric contribution from UPF in the diet of Brazilian women in social vulnerability. Maceió, 2020/2021 (*n* 1702)

	Food addiction					
	Crude			Multivariate^*^		
Variables	OR	IC 95 %	*P*-value	OR	IC 95 %	*P*-value
Availability of UPF in the food environment^†^						
T1	1·00			1·00		
T2	1·19	0·82, 1·72	0·36	1·21	0·83, 1·76	0·31
T3	1·44	1·02, 2·05	0·04	1·53	1·07, 2·18	0·02
Energy contribution from UPF^‡^						
T1	1·00			1·00		
T2	1·27	0·91, 1·76	0·15	1·32	0·92, 1·88	0·13
T3	1·36	0·97, 1·90	0·06	1·39	1·48, 1·97	<0·01

UPF, ultra-processed foods.

The modified Yale Food Addiction Scale 2.0^(20)^ was used to assess food addiction.

* Analysis adjusted for age, race/skin colour, monthly per capita family income and reported medical diagnosis of anxiety and depression.

† Through the audit of retailers with the support of the AUDITNOVA instrument^(31)^, the availability of UPF in the consumer food environment was assessed^(6)^.

‡ Through the application of 24-hour dietary recall, dietary intake was assessed, and subsequently, the energetic participation from UPF in the diet, taking into account the NOVA classification of foods proposed by Monteiro et al.^(26)^.

## Discussion

Our findings demonstrate that women living in situations of social vulnerability inserted in a food environment characterised by high UPF availability are 1·5 times more likely to present FA. At the same time, we also observed that the high caloric contribution from UPF increased up to 1·4 times the chance of these women presenting FA.

The proportion of FA (14·6 %) found in this study is higher than that observed in other studies conducted in several countries. In studies that also used mYFAS 2.0, it was possible to observe that women evaluated the prevalence of FA at 5·3 % in Brazil^(20)^ and 14·26 % in the United States^(29)^. Using the Yale Food Addiction Scale 2.0^(32)^, other studies identified FA prevalence rates of 11·9 % in Denmark^(33)^. However, it is worth noting that the studies cited were not carried out in locations characterised as socially vulnerable, demonstrating that the population living in these places may be more affected by FA.

In another study conducted with low-income women of reproductive age living in southeast Texas, United States, it was possible to identify a prevalence of FA through the Yale Food Addiction Scale of 2·8 %^(34)^. However, it is noteworthy that in that study, almost 60 % of the sample had an annual income of up to $15 000·00 per family. This situation demonstrates their greater economic favorability compared with the population included in our study.

With regard to the number of FA symptoms, the average found in this study, 1·06 (1·9), is higher than that found in another study carried out in Michigan, United States, and also of low-income women 0·6 (1·3)^(35)^. However, as highlighted above, when comparing the low-income situation between Brazil and the United States, it is possible to highlight that the population included in this study has a much more unfavourable economic situation.

Thus, evaluating the presence of FA in the low-income population, as in our study, demonstrates relevance because the difficulty in access to financial resources can favour a more stressful environment, leading to a greater need to save in essential areas such as food, affecting the greater acquisition and consumption of foods of low nutritional quality and greater palatability, such as UPF^(36)^. This situation is all the more worrying because processed and UPF foods in Brazil, according to price projections, have become cheaper than unprocessed or minimally processed foods since 2023, favouring their increased consumption, especially among the poorest people^(37)^.

Our findings also show that the high availability of UPF in the food environment increases the chances of FA by 47 %. This finding is worrying; given that in another study conducted in Brazilian favelas, it was possible to identify that establishments offering predominantly healthy foods are less numerous and more distant from the favelas, while those offering predominantly unhealthy foods are widely available in the environment^(38)^.

These data corroborate the environmental results presented in this study. They also present a panorama similar to that discussed in another study by our research group, which more broadly showed the high availability of UPF and the low healthiness of the food environment in the favelas of Maceió^(3)^. In this same study, we identified that the population’s perception of the healthiness of the food environment in the favelas was low, possibly as a result of the high availability of UPF. We also found that the worst perception of the environment was related to a greater chance of being overweight and abdominal obesity and that low healthiness also increased the chance of abdominal obesity^(3)^, situations that favour FA among women.

A systematic review that evaluated studies with populations of low socio-economic status in the USA identified the presence of adverse health outcomes in this population as a result of greater access to convenience stores and fast food^(39)^. Knowing that even the poorest populations in the USA have higher incomes than those in Brazil, the food environment in Brazilian favelas is even more worrying^(3)^, as it may be associated to a greater extent with adverse health outcomes in the population living there, as well as favouring FA even more alarmingly.

Another extremely important point relates to the context of violence and social inequality in the areas assessed^(40)^. Other equally important points concern the lack of structure and access to essential public services^(41)^. These issues can directly influence food consumption. This panorama means that this population, in addition to FA, is also more exposed to the occurrence of chronic non-communicable diseases due to the profile of the food environment that enables greater access to UPF^(42)^.

In addition, it is noteworthy that the food environment is directly related to the consumption profile of the population, and it is possible to conclude that the increased availability of UPF in the food environment favours the increase in its consumption^(4)^. In this direction, our findings demonstrate that the high caloric contribution of UPF, due to increased consumption, is associated with a higher occurrence of FA. This situation may increase eating disorders. The relationship between the consumption of UPF and FA has been identified previously, with Australian young adults with FA tending to consume more of them compared to those without this condition^(5)^.

In addition to contributing to the increase in the prevalence of FA, the consumption of UPF also favours weight gain, noticing that individuals with obesity have a higher consumption of this type of food^(43)^. It is also worth noting the positive relationship between FA and the diagnosis of chronic non-communicable diseases, such as type 2 diabetes mellitus^(44)^. When looking specifically at type 2 diabetes mellitus, there is also a direct relationship with the consumption of UPF^(45)^. Interventions aimed at reducing the consumption of foods rich in carbohydrates, one of the characteristics of UPF, even in a short period of treatment, point to an improvement in the panorama related to FA, being identified as the reduction of symptoms presented before starting the intervention^(46)^, besides contributing to weight loss^(47)^.

However, this is not an easily achieved condition since individuals with FA present compensatory stimuli similar to those with illicit substance use disorder^(48)^. This condition is observed because overeating due to FA leads to changes in the neural reward system, triggering a dysregulation of the dopaminergic system. This situation causes the uncontrollable desire to eat foods that give more pleasure, in general, those that are high in sugar, fat and Na, as the UPF^(49)^ that were associated with FA among vulnerable women in this study.

This study presents some strong points, among which, to the best of our knowledge, it was the first work to evaluate the relationship between the food environment and FA, adding new evidence to the subject studied, a situation that is pertinent due to the influence that has been perceived between this environmental component and the eating pattern of the Brazilian population^(4)^, also, for being a population-based study conducted in a representative sample of adult women of reproductive age living in a situation of social vulnerability in a Brazilian capital. Because all evaluations were conducted in person in the participant’s households by trained and standardised interviewers, using validated and reliable instruments to assess the outcome and independent variables may have directly influenced the quality of the collected data.

The present study also presents some limitations, such as the transversal design that weakened the establishment of causal inferences. Also, the self-report of previous diagnosis of depression, which may have induced the participants not to answer what corresponds to reality, and also the possible embarrassment of the interviewees to admit the occurrence of any of the symptoms described in mYFAS 2.0, a condition that we tried to minimise by conducting the interviews in spaces where there was no presence of other people. The study was also carried out during the COVID-19 pandemic, which may have had an even greater economic impact on the population studied. However, taking into account the financial aid offered by the Brazilian government to the poorest population, as well as the support network formed especially by society, this aspect has been minimised, as observed in Silva-Neto et al.^(50)^ study carried out less than a year after the first case of COVID-19 was confirmed in Brazil.

In conclusion, we can observe that the food environment, about the greater availability of UPF, is a factor associated with a higher occurrence of FA in women living in social vulnerability and higher consumption of UPF. Thus, discussing the role of the food environment in the consumption of UPF and addictive behaviour is fundamental to the health and nutrition agenda.

The evidence presented here, especially regarding the food environment, indicates a new line of investigation that should be considered to formulate strategies that focus on decreasing the prevalence of FA, especially in women with higher consumption of UPF. It also demonstrates the need for more urgent implementation of measures aimed at reversing the current profile of the food environment in Brazil’s favelas. This requires medium- and long-term measures, such as government incentives for the establishment of food retail aimed at providing greater access to healthy food. However, in order for the population to be able to purchase these foods, it is necessary to assess issues related to reducing the tax burden on healthy foods. This condition can be subsidised by surcharging unhealthy foods, such as UPF.

There is also a need to intervene in more complex situations related to access to food. These issues range from individual aspects, such as job opportunities for women, especially black women, to elements of the microenvironment, such as violence, lack of basic sanitation and health services and the macroenvironment, such as racism and food production and distribution. In view of this, the complexity of changing the scenario presented here is evident, demonstrating the need to formulate public policies that integrate the most different government sectors in order to reverse the panorama highlighted in this study.

## Supporting information

Silva-Neto et al. supplementary material 1 Silva-Neto et al. supplementary material

Silva-Neto et al. supplementary material 2 Silva-Neto et al. supplementary material

## Supplementary material

For supplementary material accompanying this paper visit https://doi.org/10.1017/S1368980025100426
