# Supplementary material for: Food environment and consumption of ultra-processed foods influencing food addiction in socially vulnerable women in Brazil
Source: Public Health Nutr. 2025 Jun 3;28(1):e106. doi: 10.1017/S1368980025100426 (PMC12264777; doi:10.1017/S1368980025100426)
Supplement: Silva-Neto et al. supplementary material 1 — Silva-Neto et al. supplementary material [file S1368980025100426sup001.docx]

Supplementary material - Flowchart of the selection of the Favelas and Urban Communities included in the study.
