# Supplementary figures and images for: Food environment and consumption of ultra-processed foods influencing food addiction in socially vulnerable women in Brazil
Source: Public Health Nutr. 2025 Jun 3;28(1):e106. doi: 10.1017/S1368980025100426 (PMC12264777; doi:10.1017/S1368980025100426)

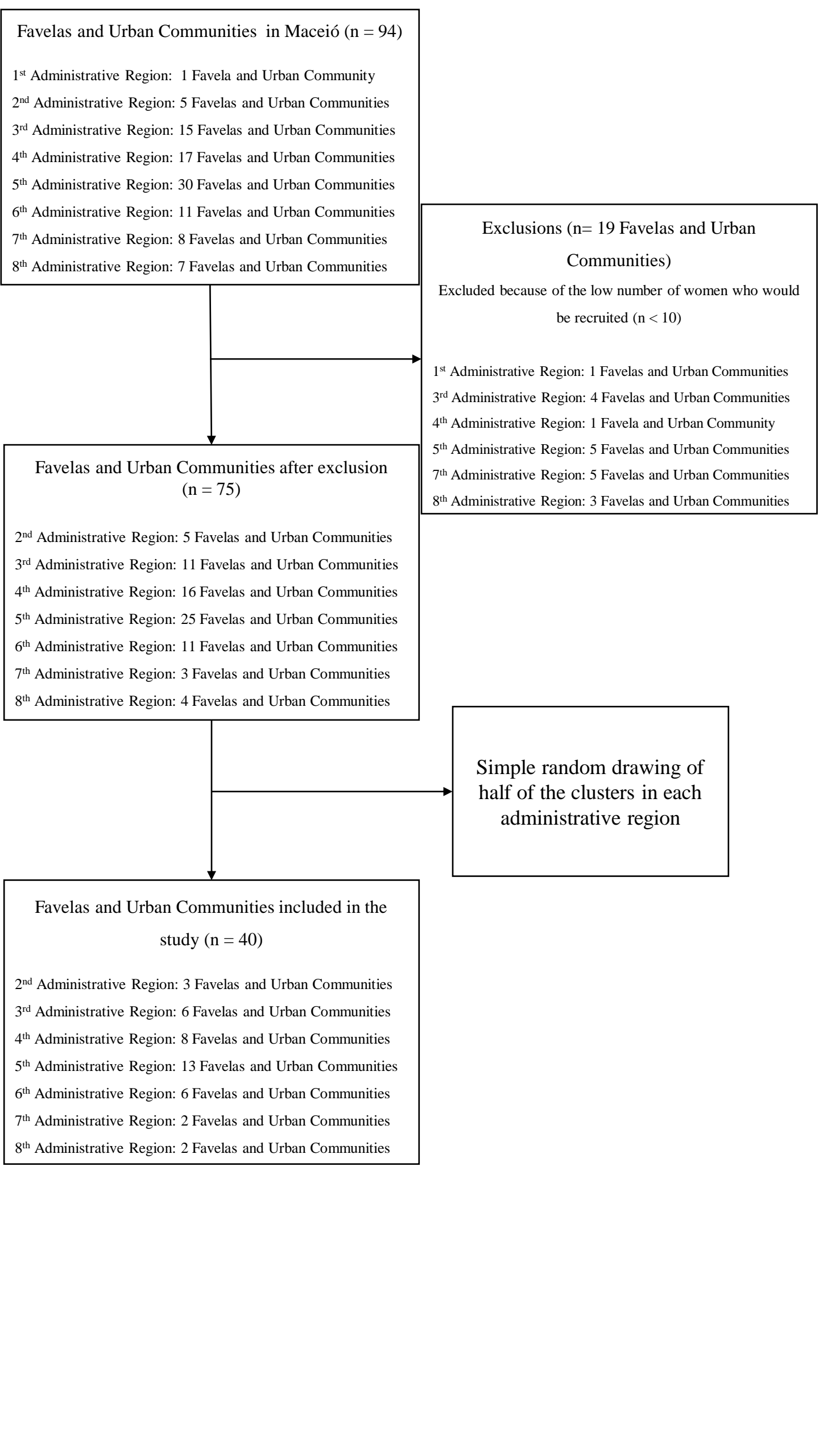

Supplement: Silva-Neto et al. supplementary material 2 — Silva-Neto et al. supplementary material [file S1368980025100426sup002.pdf]
